# The use of bioactive peptides to modify materials for bone tissue repair

**DOI:** 10.1093/rb/rbx011

**Published:** 2017-04-16

**Authors:** Cunyang Wang, Yan Liu, Yubo Fan, Xiaoming Li

**Affiliations:** 1 Key Laboratory for Biomechanics and Mechanobiology of Ministry of Education, School of Biological Science and Medical Engineering, Beihang University, Beijing 100191, China;; 2 School of Aeronautic Science and Engineering, Beihang University, Beijing 100191, China;; 3 Key Laboratory of Advanced Materials of Ministry of Education of China, Tsinghua University, Beijing 100084, China

**Keywords:** bone repair material, peptide, osteogenic activity

## Abstract

It has been well recognized that the modification of biomaterials with appropriate bioactive peptides could further enhance their functions. Especially, it has been shown that peptide-modified bone repair materials could promote new bone formation more efficiently compared with conventional ones. The purpose of this article is to give a general review of recent studies on bioactive peptide-modified materials for bone tissue repair. Firstly, the main peptides for inducing bone regeneration and commonly used methods to prepare peptide-modified bone repair materials are introduced. Then, current *in vitro* and *in vivo* research progress of peptide-modified composites used as potential bone repair materials are reviewed and discussed. Generally speaking, the recent related studies have fully suggested that the modification of bone repair materials with osteogenic-related peptides provide promising strategies for the development of bioactive materials and substrates for enhanced bone regeneration and the therapy of bone tissue diseases. Furthermore, we have proposed some research trends in the conclusion and perspectives part.

## Introduction

Recently, the biomaterials community has increasingly embraced the concept that implanted substrates should not only provide structural support for damaged tissues, but also integrate with the surrounding tissues and promote the desired tissue regeneration [1–3]. In order to achieve this goal, researchers have been not only trying their best to choose appropriate raw materials and utilize advanced preparation methods, but also making great efforts to further functionalise the materials with effective optimizing techniques. Up to now, a large panel of natural and synthetic materials has been investigated for bone tissue engineering applications. However, no single material fulfils all the criteria of biocompatibility and bioactivity [4]. Although it was previously thought that materials should present a relatively biotolerant surface in order to minimize immune and fibrotic responses, more and more evidence now suggests that interactive, biomimetic surfaces often exhibit enhanced performances [5]. For instance, the efficacy of osseointegration is mostly dependent on the interactions between the implants and osteogenic macromolecules in blood [6]. Some biomaterials do not readily adsorb blood proteins to their surface, and therefore do not support well bone-related cell activities, potentially leading to limited bone formation. Consequently, these materials need to be further optimized to enhance and accelerate bone ingrowth [7]. The successful development of high-performance biomaterials must take into consideration how their surfaces will interact with *in vivo* substances, which has prompted a burgeoning effort to engineer materials with biomolecules or modify their surfaces with biologic elements [8, 9]. One common approach is to functionalise biomaterial surfaces with osteoinductive molecules, including growth factors or peptides [4]. For the design of biomaterials, it has been well recognized that desired cell responses are crucial [10]. The immobilization of certain molecules on scaffolds has been shown very effective to improve not only recruiting stem cells but also triggering their osteogenic differentiation because these molecules can provide osteogenesis-stimulating signals for the cells [4, 11]. The practical advantage of using peptides, rather than native proteins, for biomaterial surface functionalization, is due to the fact they can be produced synthetically with precise control of their chemical composition, thereby avoiding pathogenic contamination from animal sources. In general, peptides are more resistant than high-molecular weight proteins, with respect to the denaturation caused by pH or temperature variations. Moreover, they are easier to manipulate during the grafting procedure. Especially, the research for bioactive peptides in orthopaedics is growing rapidly in recent years.

In this article, we have reviewed recent studies on bioactive peptide-modified materials for bone tissue repair. Firstly, the main peptides for inducing bone regeneration and commonly-used methods to prepare peptide-modified bone repair materials are introduced. Then, current *in vitro* and *in vivo* research progress of peptide-modified composites used as potential bone repair materials are reviewed and discussed. Furthermore, the related research trends are proposed in the conclusion and perspectives part.

## The main peptides used for bone tissue repair

In the past two decades, a variety of bioactive peptides have been studied and applied for the promotion of bone regeneration to repair local bone defects or treat other bone diseases. In this section, we divided these peptides into three categories, including extracellular matrix (ECM)-derived peptides, bone morphogenetic proteins (BMPs)-derived peptides and others, and respectively introduced and discussed the current progress of investigations into them as follows.

### ECM-derived peptides

ECM is an aggregation of extracellular molecules from different cells to provide biochemical and structural supports [12]. The ECM-derived peptides with signalling domains, which are capable of connecting with receptors on the surface of cell membrane, is a hotspot of research. Variety of ECM-related peptides shown in Table 1 have been studied for bone tissue repair and regeneration [13].

**Table 1.** *In Vitro* and *in vivo* studies presenting the osteogenic effects of ECM-derived peptides

Active peptides	Abbreviation	Composition	Binding sites	Upregulation / Downregulation of proteins or genes	Final functions	References
PepGen P-15	P-15	15 amino acids	type I collagens-binding sites	upregulating the expression of ALP, BMP-2, BMP-7; RUNX2, COL1, OSTRX and BSP; a2 integrin	upregulating proliferation and osteogenic differentiation; cell attachment, migration and survival; extracellular matrix production	14–16, 20
arginyl-glycyl- aspartic acid	RGD	3 amino acids	integrin-binding sites	upregulating the expression of ALP, RUNX2, osteocalcin, osteopontin and BSP; Sox9, Aggrecan, fibronectin and clloagen II	upregulation proliferation; osteogenic differentiation and mineralization; cell attachment and survival	21–25
Ser-Val-Val-Tyr- Gly-Leu-Arg	SVVYGLR	7 amino acids	RGD-binding sites	upregulating αvβ3 integrin; suppressing NFAT activity and expression of osteoclastogenesis-related mRNAs	upregulating proliferation and neovascularization; angiogenesis and osteogenesis; adhesion, migration and tube formation of endothelial cells	26–28
glycine-phenylalanine- hydroxyproline-arginine	GFOGER	4 amino acids	α2β1 integrin-binding sites	upregulating α2β1 integrin binding	upregulating differentiation, bone regeneration and osseointegration	29–32
Asp-Gly-Glu-Ala	DGEA	4 amino acids	α2β1 integrin-binding sites	upregulating ALP	cell adhesion, spreading and osteogenic differentiation	33–37
collagen-binding motif	CBM	28 amino acids	collagen-binding sites	inducing sustained activation of ERK; the transactivation of SRE, CRE, and AP-1; expression of type X collagen	upregulating bone-related cell adhesion and growth; osteogenic differentiation	38–39
lysine-arginine- serine-arginine	KRSR	4 amino acids	heparin-binding sites	upregulating osteogenic gene expression	upregulating bone-related cell spreading, adhesion and mineralization	40–46
Phe-His-Arg-Arg- Ile-Lys-Ala	FHRRIKA	10 amino acids	putative heparin-binding sites	–	upregulating bone-related cell spreading, adhesion and mineralization	47, 48
Fibronectin-derived peptides	FN-derived peptides	7 amino acids	–	–	upregulating bone-related cell spreading, adhesion and mineralization	49–51

The PepGen P-15 (P-15) is a kind of peptide containing 15 amino acid sequences (766-GTPGPQGIAGQRGVV-780) and identical to the cell-binding region of type I collagens [14]. Its capacity of supporting bone growth is approved by several studies, such as stimulating osteoblast proliferation and differentiation by enhancing cell attachment to bone repair materials and upregulating ECM production [15, 16]. Different studies have shown that the P-15 could increase not only osteogenic gene expression but also the expression of osteogenic alkaline phosphatase (ALP) proteins and matrix mineralization due to upregulating runt-related transcription factor-2 (RUNX2), collagen 1(COL1), osterix (OSTRX) and bone sialoprotein (BSP) [17–19]. Furthermore, in the study of Nguyen *et al.* [20], they found that P-15 could significantly enhance the gene expression of BMP-2 and BMP-7 in human osteosarcoma cell (HOS).

The arginyl-glycyl-aspartic acid (RGD) peptide, which is comprised of 3 amino acid residues, is the cardinal integrin-binding domain and presents in many extracellular matrix proteins, such as fibronectin and vitronectin [21, 22]. As a part of cell surface signalling, RGD peptide can enhance the expression of ALP, Runx2, osteocalcin (OCN), osteopontin (OPN) and BSP to ensure osteoblast proliferation, differentiation and mineralization. For example, Huang *et al.* found that RGD increased cell attachment, and enhanced cellular proliferation [23]. Furthermore, Rammelt and his group further used RGD peptide to enhance the adhesion and growth of cells [24, 25].

The Ser-Val-Val-Tyr-Gly-Leu-Arg (SVVYGLR) peptide that are adjacent to RGD sequence in osteopontin can not only significantly improve the proliferation of MSCs, but also upregulate neovascularization [26–28]. The relative results can be found in the studies of Egusa *et al.* [27] and Hamada *et al.* [28] who investigated bioactive function of a SVVYGLR peptide in osteoclasts and osteoprogenitor cells.

The GFOGER (glycine-phenylalanine-hydroxyproline-arginine) as a collagen-mimetic peptide can improve osteogenic differentiation, bone regeneration and osseointegration [29–31]. In the study of Shekaran *et al.*, they showed that GFOGER could accelerate and increase bone formation in the femoral defect model [29–31]. Moreover, Mhanna *et al.* showed that GFOGER played a crucial role in the proliferation and osteogenic differentiation of mesenchyal stem cells (MSCs) [32].

The Asp-Gly-Glu-Ala (DGEA) that is a kind of collagen peptide can bind to α2β1 integrin, which is an extracellular matrix receptor for collagens and/or laminins, and promote cell adhesion, spreading and osteogenic differentiation [33]. The study of Hennessy *et al.* showed that DGEA stimulated osteogenic differentiation of MSCs [34]. In the study of Ceylan *et al.*, they synthesized peptide nanofibers with DGEA to promote the osteogenic differentiation of progenitor stem cells and bone-like mineralization [35]. Moreover, it was shown that DGEA peptide enhanced adhesion and osteogenic differentiation of bone marrow stem cells [35–37].

As a cleavage product of osteopontin, the collagen-binding motif (CBM) can promote osteogenic differentiation and migration through some specific signalling pathways [38, 39]. For example, the evidence provided by the study of Shin *et al.* indicated that CBM could promote osteogenic differentiation through Ca^2+^/CaMKII/ERK/AP-1 signalling pathway in hMSCs. Furthermore, they also found that CBM stimulated the migration of hMSCs by suppressing cell proliferation [38].

In bone sialoprotein, vitronectin, fibronectin, osteopontin and thrombospondin, researchers found that lysine-arginine-serine-arginine (KRSR) as a heparin-binding site could increase osteogenic gene expression and osteoblast adhesion [40–44]. Moreover, in the study of Dettin *et al.* [45] and Hasenbein *et al.* [46] it was shown that KRSR motif was selective for osteoblast attachment, not for endothelial cells or fibroblasts. In addition, they also found that KRSR could enhance osteoblast spreading.

The Phe-His-Arg-Arg-Ile-Lys-Ala (FHRRIKA) is a putative heparin-binding domain of bone sialoprotein. It can enhance the ability of osteoblast adhesion, spreading and mineralization [47]. In the work of Gentile *et al*, they showed that FHRRIKA could induce cell adhesion and bone mineralization [48].

Fibronectin (FN)-derived peptides could improve osteoblast adhesion, spreading and mineralization [49]. Lee *et al.* used model of rabbit calvarial defect to find new bone formation enhanced by a fibrin-binding synthetic oligopeptide derived from FN [50]. In addition, Martino *et al.* reported the regenerative effects of FN III9-10/12-14 on enhancing platelet-derived growth factor-BB (PDGF-BB) and BMP-2 in a critical-size bone defect model [51].

### BMPs-derived peptides

BMPs are a group of growth factors to induce the formation of bone or cartilage [52]. Bone-repairing responses of BMPs-derived peptides have been widely studied by scholars [53]. These peptides have been mainly derived from BMP-2, BMP-7 and BMP-9 to promote bone-repairing responses. BMP-2-derived peptide as residues of BMP-2, including P17, P20 and P24, can regulate bone-repairing responses [4, 54–59]. The effect of BMP-2-derived peptide on bone tissue repair and regeneration as a key point is studied. For example, Zhang *et al.* found the synthetic peptides derived from BMP-2 residues 32-48 (P17-BMP-2) could significantly upregulate bone-repairing response [60]. In the study of Zhou *et al.* residues 73-92 of BMP-2 was used to induce osteogenic differentiation and bone regeneration [61]. The study of Kim *et al.* [62] also revealed the positive effects of BMP-2 peptides on osteogenic differentiation of hMSCs. Moreover, Lin *et al.* [63] synthesized BMP-2-derived peptide, P24, and found that it could promote osteogenic differentiation of BMSCs.

In order to discover the functions of other BMPs-derived peptides to stimulate bone formation, Kim *et al.* [64] found a new BMP-7-derived peptide, BFP-1. By adding BFP-1 into culture medium of BMSCs, they found that BFP-1 could enhance Ca^2+^ content in the cells and induced their ALP activity. In the study of Bo *et al.* [65], they used BMP-7 mimetic peptide to accelerate bone regeneration.

Moreover, Lord *et al.* [66] used human white preadipocytes (HWP) to determine the effects of BMP-9-derived peptide, pBMP-9, and found that the pBMP-9 did not affect the number of apoptotic cells along with reducing the proliferation of HWP. They also found that the pBMP-9 inhibited the proliferation and induced osteogenic differentiation of preosteoblasts when its content was about 400 ng/ml.

### Other peptides

In addition to the peptides derived from ECM and BMPs, as shown above, other ones shown in Table 2 have also been studied and developed to induce bone regeneration.

**Table 2.** *In Vitro* and *in vivo* studies presenting the osteogenic effects of other peptides except those derived from ECM and BMPs

Active peptides	Abbreviation	Composition	Potential pathways	Upregulation / Downregulation of proteins or genes	Final functions	References
calcitonin gene-related peptides	CGRP	37 amino acids	the cAMP, Wnt and AMPK-eNOS pathways	upregulating expression of IGF-1, IGF-1 receptor and BMP-2 receptor; ALP, OC and COLLA1	upregulating cell proliferation, osteogenic differentiation and angiogenesis; downregulating apoptosis and inflammation	67-85
Parathyroid hormone (1-34)	PTH_1-34_	34 amino acids	G(q)-signalling, β-arrestin recruitment, ERK1/2 phosphorylation and phospholipase C pathway	upregulating expression of Runx2 and COL2A1; downregulating expression of ALP and BMP-2	upregulating cell proliferation and chondrogenesis	86-88
osteogenic growth peptides	OGP	14 amino acids	the G1 protein-MAPK and RhoA/ROCK pathway	upregulating osteocalcin, collagen, BMP-2, ALP and mineralization; upregulating TGF β1, β2, β3, FGF-2, IGF-I	upregulating cell proligeration and osteogenic differentiation; cartilage-to-bone transition; downregulating adipogenic differentiation	89-96
thrombin peptide 508	TP508	23 amino acids	cell cycle-G1/S checkpoint, JAK/STAT, NF-kappaB, PDGF, PI3K/AKT, PTEN, and ERK/MAPK	upregulating the expression of Runx2 and OPN	upregulating cell proliferation and osteogenic differentiation; chemotaxis, angiogenesis and revascularization; downregulating apoptosis, the effect of hypoxia	22, 97-99
NEMO-binding domain peptide	NBD	6 amino acids	NF-κB pathway	downregulating TRAP activity, actin rings; RANKL-induced c-Src kinase activity	upregulating osteogenic differentiation of cells; downregulating bone resorption	100-103
Cell penetrating peptide	CPP	30 amino acids	–	–	transcriptional factor to deliver bone-regenerating related proteins or factors into cells	104-106
AcN-RADARADARADARADA-CONH_2_	RADA16-I	16 amino acids	–	upregulating expression of Runx2 genes, ALP and osteocalcin	transcriptional factor to deliver bone-regenerating related proteins or factors into cells	107-109

The calcitonin gene-related peptide (CGRP) has been widely studied due to their positive effects on bone-repairing response. CGRP is discovered in bone marrow, metaphysis and periosteum, etc. Two different forms, CGRP-α and CGRP-β, are derived from separate genes [67]. Compared with CGRP-β, CGRP-α can enhance osteoblast proliferation and bone regeneration [68–71]. The previous studies showed that CGRP could not only enhance osteoblast proliferation and differentiation because it could bond with functional receptors and transporters on the bone-related cells, but also stimulate the production of growth factors, including BMP-2 and IGF-1. In addition, the CGRP could reduce the apoptosis and inflammation [72–83]. Its positive effects on the bone-related cells and new bone formation can be found in many studies. For instance, Lv *et al.* found that CGRP was an important factor and could enhance bone density to improve the quality of regenerated alveolar bone [84]. Zhou *et al.* elucidated the important role of CGRP by spinal fusion model, and found that expression of CGRP was located around the interface between allograft and fibrous tissue to induce the osteogenic differentiation of cells and new bone formation [85].

With 34 amino acids, the peptide derived from parathyroid hormone (PTH_1-34_) is one of the earliest artificially synthesized amino acid fragments [86]. It was shown that the PTH_1-34_ peptide could upregulate proliferation and osteogenic differentiation of cells by enhancing the expressions of Runx2 and OPN. Alkhiary *et al.* used animal models to demonstrate that PTH_1-34_ could improve bone regeneration by enhancing bone density, mineral content and strength [87]. In addition, it could also increase angiogenesis and revascularization [88]. Therefore, PTH_1-34_ is one of effective peptides for the improvement of fracture-healing.

As H4 histone-related peptide, osteogenic growth peptide (OGP) is highly conserved with 14 amino acids, which was found in serum [89, 90]. It could activate an intracellular Gi-protein-MAP kinase signalling pathway [91, 92]. OGP have been found to increase the proliferation, osteogenic differentiation and matrix mineralization of bone-related cells [93–96]. Brager *et al.* showed that OGP regulated TGF-β1, β2, β3, IGF-I, FGF-2 and aggrecan *in vivo* [93]. Bab *et al.* found OGP increased overall bone mass and bone formation quality by exerting an anabolic effect on bone cells [89].

With 23 amino acids, thrombin peptide 508 (TP508) is a synthetic peptide that represents the non-proteolytic receptor binding domain of thrombin. TP508 enhances not only proliferation and osteogenic differentiation of human osteoblasts, but also increased angiogenesis [97, 98]. For instance, Hanratty *et al.* [99] improved healing effect of murine fracture by injecting the TP508 peptide into the fracture gap. The similar result can be found in the study of Cakarer *et al*., which showed that the TP508 peptide could enhance bone consolidation in tibial distraction osteogenesis [22].

Kappa-B kinase (IKK) is an inhibitor of nuclear factor with two catalytic subunits, IKK-1 and IKK-2. Strnad *et al.* reported it was a non-catalytic regulatory subunit NF-kB essential modulator (NEMO or IKK-γ), and the NEMO-binding domain (NBD) is an interacting region with 6-amino acids where IKK subunits interacts with NEMO [100, 101]. Furthermore, the studies of Li *et al.* [102] and Jimi *et al.* [103] showed that this peptide could induce osteogenic differentiation of cells and inhibit bone resorption.

Cell penetrating peptides (CPPs) can transport various molecules into the cytoplasm through cell membranes [104, 105]. With the above properties, CPPs can behave as a transcriptional factor to deliver bone-repairing relevant proteins or factors into cells. For example, in a calvarial defect model, Park *et al*. used the CPPs to transfer recombinant adenovirus expressing BMP-2 into MSCs to promote new bone formation [106].

RADA16-I (AcN-RADARADARADARADA-CONH_2_) is generated by spontaneous assembly of several fragments into ordered nanostructures [107]. The studies of Li *et al.* [108] and Hou *et al.* [109] showed that MSCs could express higher level of Runx2 genes, ALP and osteocalcin proteins in demineralized bone matrix (DBM) modified with RADA16-I compared to the bare DBM.

## The commonly-used methods to prepare peptide-modified bone repair materials

The preparation method of peptide-modified bone repair materials determines the manner, in which the peptide is bonded to the substrate material, and has a significant effect on the final osteogenic activity of the composite. Currently, the commonly-used preparation methods include electrodeposition, covalent immobilization, physical adsorption and others.

### Electrodeposition

Traditionally, electrodeposition is an electrochemically process, during which a material is deposited from its compound aqueous solution, non-aqueous solution or molten salt. It is necessary that both positive and negative poles should be inert because the cations in the solution need to be introduced to the cathode. It has been shown that this technique has been used to compound peptides with artificial biomaterials. In order to prepare peptide-modified materials by electrodeposition, we need to take the following steps. At first, the terminally modified polymers, containing both –NH_2_ and –COOH at terminals, should be fabricated. Then, the polymers are dissolved in the electrolyte solution (*eg.* NaCl, PBS, etc.), in which –${\text{NH}}_{3}^{+}$ and –COO^–^ are formed on the polymers by hydrolysis of –NH_2_ and –COOH. And then, –${\text{NH}}_{3}^{+}$ of the polymers are combined with –OH^-^ of cathode by an ionic bond, N–HO under the action of an electric field. Finally, the materials are immersed in an aqueous solution containing peptides where –${\text{NH}}_{3}^{+}$ of the peptides are connected with –COO^–^ of the polymers through an ionic bond, resulting in the immobilization of the peptides on the materials.

**Figure 1.**
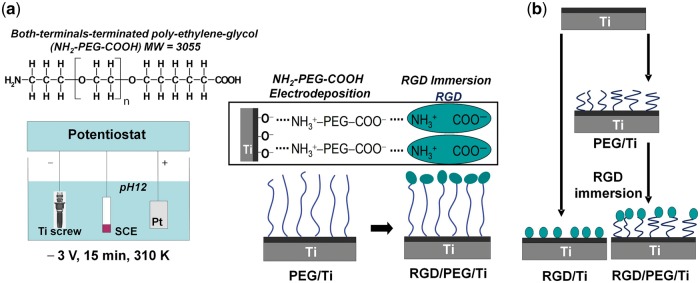
(a) Process illustration of covalent immobilization of the RGD peptide on Ti bone repair material by electrodeposition, (b) simple description of the difference between RGD/PEG/Ti and RGD/Ti materials (adapted with permission from ref. [17]. Copyright 2011 Elsevier Ltd)

Oya *et al.* [18] fabricated RGD peptide-modified titanium (Ti) materials by electrodeposition. At first, poly(ethylene glycol) (PEG) was terminated with two kinds of functional groups, –NH_2_ and –COOH, and the NH_2_–PEG–COOH was dissolved in a 0.5 mol/l NaCl solution with a concentration of 0.2 mass%. Meanwhile, glycine (Gly) with both –NH_2_ and –COOH was selected as a control, which was dissolved in the same solution with a concentration of 0.0076 mass%. They found that the polymer could be most effectively electrodeposited when pH of the solution was adjusted to 12. Under the action of the electric field 3.0 V for 900 s at 37°C, the NH_2_–PEG–COOH and glycine respectively migrated to the cathode Ti and were immobilized on it by an ionic bond. Then, each specimen was immersed in a 0.001 mass% RGD aqueous solution with pH 12 at 37°C for 24 h. After immobilization, the specimens were rinsed and dried. The chemical structure formulae of NH_2_–PEG–COOH and schematic illustration of RGD immobilization on the Ti implant was shown in Fig. 1. The thickness of the immobilized layer on Ti of each specimen was determined by ellipsometry, which showed that more RGD molecules were immobilized on PEG/Ti than on Gly/Ti, indicating that PEG is a kind of satisfactory polymer for preparing peptide-modified materials by electrodeposition.

Moreover, it has been shown that the surface morphology of the materials was obviously changed during the whole electrodeposition, which was observed by Park *et al.* with scanning probe microscopy [17]. The nano-scale clumps could be observed when only PEG or RGD was introduced on Ti. However, when RGD was immobilized on PEG/Ti, the surface became smooth.

Above all, it is obvious that the deposition of the polymers on the cathode is very important for the successful preparation of the peptide-modified materials by electrodeposition. In this process, it has been shown that the terminals of polymer and the pH of the electrolyte have significant effects on the deposition efficiency of the polymers, which is bound to determine the final bonding results of peptides. Tanaka *et al.* electrodeposited two kinds of PEG polymers terminated with different chemical groups on titanium oxide, and characterized the deposition efficiency by x-ray photoelectron spectroscopy (XPS) with an angle-resolved technique and glow discharge optical emission spectroscopy (GD-OES), which showed that the polymers with two kinds of terminal groups, –NH_2_ and –COOH, could be deposited on the substrate more stable than those with only one terminal group [110, 111]. They also studied the effects of the pH of the electrolyte on the deposition efficiency of the PEG polymer with two kinds of terminal groups, –NH_2_ and –COOH. They selected five different pH values, 2, 4, 7, 10 and 12. Their final results showed that when the pH of the electrolyte was 12, electrostatic reactivity between the polymer and substrate was the highest and the thickness of the polymer layer was the largest. The terminals of PEG were oriented perpendicular to the substrate and formed stable U–shape immobilization [112]. Conformational changes in the adsorbed RGD peptides are dependent on the surface energy and nanotopography of the Ti surface. The RGD was more firmly immobilized on the materials through PEG terminated with two kinds of functional groups.

### Covalent immobilization

The preparation of peptide-modified materials by covalent immobilization generally requires the chemical reactions between functional groups on substrate materials and aminos or carboxies of peptides. This process normally includes two steps. Firstly, some specific functional groups need to be introduced to the substrate materials. Then, the substrate materials are immersed into the peptide solution with or without ultrasonic treatment to form the covalent bonds between the substrate materials and peptides. During the process of this preparation method, a variety of different techniques have been used to achieve the above effects, which include epoxy linkages, silane couplings, polydopamine-assisted immobilization, and thiol-ene click reactions, etc. [62, 113–117].

For the preparation by epoxy linkages, there are normally two different procedures to follow. The first one is that the chemical reactions between some specific epoxy compounds and substrate materials with –COOH are launched to produce O-acylurea on the substrate materials, -C = O- of which is then made to react with –NH_2_ of the peptides to form amido bond, thereby achieving covalent immobilization of the peptides on the substrate materials. Seo *et al.* [113] fabricated RGD peptide covalently modified titanium surface by this procedure. The titanium surface, which has been modified by polyacrylic acid (PAA) to graft the carboxyl group (Ti/COOH), was immersed in 100 mg of EDC (1-Ethyl-3-[3dimethylaminopropyl] carbodiimide hydrochloride) with NHS (N-hydroxysuccinimide) mixture and 10 ml of phosphate-buffered saline (PBS) for 24 h with gentle shaking at 0–4°C to generate O-acylurea. Then, the modified materials were immersed in 0.1 mg/ml RGD solution for 36 h with mild stirring at 0–4°C to achieve a covalent bond between RGD peptide and Ti substrate. Attenuated total reflectance Fourier transform infrared spectroscopy (ATR-FTIR) showed N-H peak and the weak C = O peak of the Ti/COOH/RGD at 3400 cm^−1^ and 1700 cm^−1^ respectively, which was related to the amide carboxyl group. The strong C = O peak near 1700 cm^−1^ of the Ti/COOH substrate was related to carboxyl groups. The result showed that the –NH_2_ groups of the RGD peptide had effectively reacted with the –COOH group on the Ti/COOH surface.

**Figure 2.**
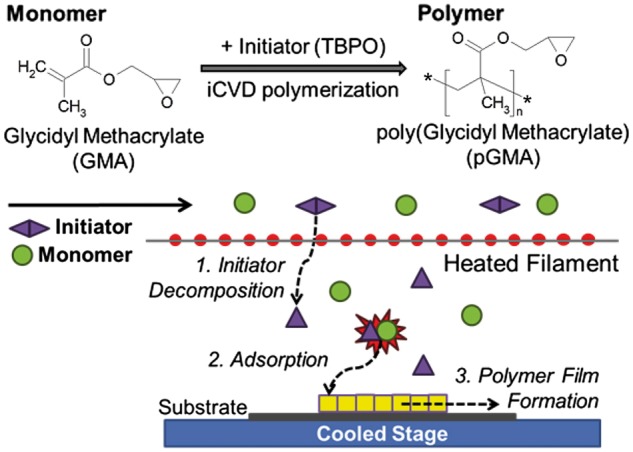
Preparation of poly(glycidyl methacrylate)-polyurethane acrylate (pGMA-PUA) nanopatterned substrate materials. pGMA was deposited onto the PUA substrates via the initiated chemical vapor deposition (iCVD) polymerization process, which was synthesized with GMA monomer and initiator (TBPO) (adapted with permission from ref. [62]. Copyright 2013 Elsevier Ltd)

The other procedure to prepare covalently modified material by epoxy linkages is that epoxy polymer are deposited on the substrate, and then their epoxy groups are made to react with –NH_2_ of the peptides to achieve covalent bonds. Kim *et al.* [62] fabricated BMP-2 peptide covalently modified nanopatterned polyurethane acrylate (PUA) substrates by this procedure. Firstly, they coated an epoxy compound, poly(glycidyl methacrylate) (pGMA) on PUA through initiated chemical vapor deposition (iCVD) technique, thereby achieving the polymerization of the epoxy compound and substrate. Then, epoxy groups of the pGMA-PUA substrates were covalently bonded with –NH_2_ of BMP-2 peptides by immersing the substrates in BMP-2 peptide solution (100 mM) for 2 h at room temperature, which is shown in Fig. 2. XPS analysis showed a strong nitrogen (N1s) atomic elemental peak at 401 eV, which confirmed that the BMP-2 peptide was successfully covalently conjugated to the substrate.

For the preparation by silane couplings, it is normally performed by means of a three-step reaction. At first, materials are silanized with silane coupling agent. Then, –NH_2_ of silylated modified materials are made to react with –COOH of a crosslinker. Finally, the outer maleimide groups of the crosslinker are made to react with the thiolgroups of the peptide. Acharya *et al.* [115] prepared matrix extracellular phosphoglycoprotein (MEPE) peptide covalently modified hydroxyapatite/β-tricalcium phosphate (HA/β-TCP) composites by this method. At first, the HA/β-TCP composite particles were soaked in a silane coupling agent, 3-aminopropyl-triethoxysilane (APTES) solution to be silanized. Then, –NH_2_ of the silanized particles were made to react with –COOH of a crosslinker, polyethylene glycol disuccinimidyl succinate (SS-PEG-SS), by soaking them in the 10 mM SS-PEG-SS solution. Finally, the modified HA/β-TCP was soaked in MEPE peptide solution to achieve the covalent reaction between the outer maleimide groups of the SS-PEG-SS and thiol groups of MEPE peptides. FTIR analysis showed that the absorbance peak of NH_2_, C = C, COO^-^, and C = O bonds increased in the MEPE peptide-immobilized HA/β-TCP, which confirmed that the MEPE peptides were successfully immobilized to the HA/β-TCP via covalent bonding.

For the fabrication by polydopamine-assisted immobilization, there are usually two steps to follow. The first step is the dopamine crosslinking on the surface of substrate materials. The cross-linking principle is that the phenolic structure of dopamine is oxidized into the quinone structure, and then crosslinked by Michael addition reactions between the quinone structure with the primary amine group of dopamine. The second step is the quinone structure of the crosslinked dopamine are reacted with the –NH_2_ of the peptide through Michael addition reactions. Pan *et al.* [116] prepared BMP-2-derived peptide (P24) covalently modified poly(lactic-co-glycolic acid (PLGA)-[Asp-PEG]_n_ scaffolds by this method. At first, the PLGA-[Asp-PEG]_n_ scaffolds were soaked in a DA solution (2 mg/mL 10 mM Tris–HCl, pH 8.5) overnight, in order to be crosslinked. Then, the scaffolds were immersed in the P24 peptide solution (1 mg/mL in10 mM Tris–HCl, pH 8.5) for 4 h, with shaking at room temperature, thereby achieving the covalent bond between the dopamine and peptide. HPLC analysis showed that this method, compared with physical adsorption, could load more peptides, and that the peptides could be released more slowly and continuously.

For the preparation by thiol-ene click reactions, at first some special polymers containing C = C need to be attached to the substrate materials by radical polymerization, and then the propionamide group on the polymers is reacted with the thiol group at the terminal of the peptide. Yang *et al.* [117] fabricated Arg-Glu-Asp-Val (REDV) peptide covalently modified polycarbonate urethane (PCU) materials by this method. Firstly, the PCU materials were immersed in a mixed solution of N-(2-hydroxypropyl)methacrylamide (HPMA) and eugenyl methacrylate (EgMA) at 30°C to achieve copolymers by radical polymerization. Then, the REDV peptide was directly covalently immobilized onto the substrate surface by a thiol–ene click reaction, which was carried out at 30°C in a nitrogen atmosphere for 30 min under the exposure of a 365 nm UVlamp (300 W) from a distance of 30 cm. The results showed the copolymer with higher EgMA content could immobilize a larger amount of REDV peptide. The result indicates that copolymer with high EgMA content contributes to the immobilization of the peptides because EgMA contains catechol.

It is obvious that peptides can be bonded to substrates firmly by this method. The modified materials have good stability. Peptides can be released slowly, the rate of which can be controlled. However, this method requires the participation of reagents with special functional groups, and the reaction mechanism is complicated.

### Physical adsorption

Physical Adsorption means that molecules or ions are attracted and attached to the surface of substrates in liquid or gas mediums by electrostatic force or Van der Waals force [118]. Using this method to prepare peptide-modified materials, we need to prepare substrates with high surface energy, and then immerse them in a supersaturated solution of the peptides. For example, Feng *et al.* [65] prepared chitosan/nano-hydroxyapatite/collagen (nHAC) composites modified by BMP-7 derived peptides by physical adsorption. Firstly, they prewetted the chitosan/nHAC composites in pure ethanol. Then, the ethanol was replaced with excess water. Subsequently, the samples were shaken continuously for 24 hours in the water. Finally the prewetted composites were impregnated with 1 mg of the BMP-7 derived peptide in 100 μL water to adsorb the peptide, followed by vacuum dried. Their subsequent *in vivo* study showed that significantly improved bone regeneration and better bone repair effectiveness were achieved with the composites loaded with BMP-7 derived peptide compared to the original composites.

In addition, Reyes *et al.* [29] coated tissue culture-treated polystyrene dishes with 300 Å of pure titanium using an electron beam evaporator, and then put 20 mg/ml GFOGER (Gly-Phe-Hyp-Gly-Glu-Arg) peptide solution (the peptide in PBS) into the dishes, which were incubated for 1 hour to make the titanium surface adsorb the peptide. Their subsequent *in vitro* study showed that the peptide treated material surface significantly enhanced osteogenic differentiation and mineralization of bone marrow stromal cells, compared to the unmodified titanium surface.

### Others

In addition to the above-mentioned methods, there are other ways to prepare peptide-modified materials for bone tissue repair, such as solvent extraction technique, molecular plasma deposition, etc.

Extraction means the separation of a substance from a mixture or solvent. In order to prepare peptide-modified materials by this technique, we need to take the following steps. The peptide is dissolved in water and the material, which is to be modified, is dissolved in an organic solvent. Then, the two solutions are mixed and emulsified to form a water/oil emulsion, which is then transferred to another aqueous phase containing an emulsifier to prepare water/oil/water emulsion. And then, the emulsion is constantly stirred to vaporize the organic solvent, which causes that the material is solidified into microspheres with the peptide inside. Finally the peptide-modified microspheres are washed and dried. For example, Hedberg *et al.* [119] fabricated thrombin peptide 508 (TP508)-modified PLGA/PEG microsphere using the solvent extraction technique. Firstly, PLGA and PEG were dissolved in 1 ml of CH_2_Cl_2_ solution. Then, 125 μl of TP508 solution was added into the PLGA/PEG solutions, and the two solutions were mixed to form a water/oil emulsion, which was then added to 1.5 ml 0.3% poly(vinyl alcohol) (PVA, an emulsifier) aqueous solution to produce a water/oil/water emulsion. And then, the water/oil/water emulsion was added to 100 ml of 0.2% aqueous isopropanol and 98.5 ml of 0.3% aqueous PVA, and stirred rapidly for one hour, which led to the formation of PLGA/PEG microspheres with the TP508 peptide inside. At last, the microspheres were separated from the solution by centrifuged at 180 × g for one min, followed by washed and dried. Their subsequent study on release kinetics indicated that the TP508 peptide-modified microspheres could release the peptide slowly and steadily, the rate of which could be well controlled by changing the preparation parameters of the microspheres.

The molecular plasma deposition (MPD) enables the deposition of uniform coatings onto substrates using corona discharge under high voltage. Peptide-modified bone repair materials can be prepared with this method by the steps as follows. First, the substrate is placed inside of a vacuum chamber, and the peptide solution is put into a reservoir, which is then added to a metallic needle. Then, the peptide solution is dispensed under a high voltage between the substrate and needle that induces a corona discharge, which then ionizes the peptide solution. And then, the ionized solution is introduced onto the substrate (positive pole) inside the vacuum chamber under the high voltage. Finally, the peptide-modified material is removed from the chamber, followed by washed and dried. For example, Balasundaram *et al.* [120] deposited arginine-glycine-aspartic acid-serine (RGDS), lysine-arginine-serine-arginine (KRSR), and isoleucinelysine-valine-alanine-valine (IKVAV) peptides on the surface of anodized titanium substrate respectively by this method. A high voltage of 20 kV was applied between the substrate and the needle. Electrospray ionization data demonstrated that the ionization process did not alter the original characters of the peptides. Their subsequent *in vitro* study showed that the peptide-modified anodized titanium substrates improved osteoblast adhesion and proliferation compared to the substrate without the introduction of the peptides.

## The current research progress of peptide-modified materials for bone tissue repair

Usually, after the preparation of bone repair materials, some *in vitro* and *in vivo* experiments need to be carried out to research its impact on cellular functions and new bone formation, thereby evaluating their osteogenic activity. In this section, we will review and discuss the current *in vitro* and *in vivo* research progress of peptide-modified composites used as potential bone repair materials.

### *In vitro* evaluations

In this subsection, we will mainly present and discuss the recent researches on the *in vitro* evaluations of peptide-modified composites used as potential bone repair materials, focusing on the effects of the materials on the functions of cultured cells *in vitro*, such as adhesion, spreading, proliferation, differentiation and mineralization, etc.

#### ECM-derived peptides modified materials

**Figure 3.**
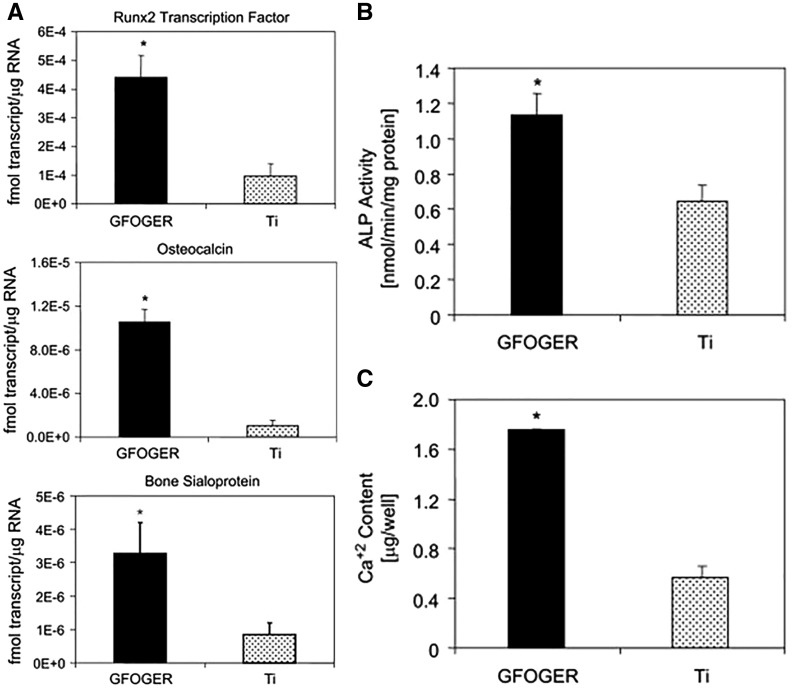
Compared to untreated titanium (Ti), GFOGER peptide coated Ti much more significantly promoted specific osteogenic gene expression (a), enhanced ALP activity (B) and biomineralization (C) of the cultured bone marrow stromal cells (adapted with permission from ref. [29]. Copyright 2007 Elsevier Ltd)

Numerous studies have indicated that the ECM-derived peptides modified materials can significantly improve the desired functions of bone-related cells *in vitro*. To date, calcium phosphate modified by ECM-derived peptides have attracted more and more attention. Hennessy *et al.* [34] studied that the efficacy of hydroxyapatite (HA) disks coated with a kind of ECM-derived peptide, DGEA, in promoting the osteogenic-related functions of MSCs, the results of which showed that cells grown on HA disks coated with DGEA (test group) exhibited greater ALP activity and OCN secretion than those on bare HA disks (control group). However, there was no obvious difference on cell adhesion between test group and control group. Hydrogel composites modified by ECM-derived peptides are another kind of materials, which have been researched widely by cell culture *in vitro* to evaluate the possibility of their application as bone repair materials. For example, Stile *et al.* [121] modified poly(N-isopropyl acrylamide-co-acrylic acid) [P(NIPAAm-co-AAc)] hydrogels with FHRRIKA peptide and studied the effects of FHRRIKA peptide addition on the cell viability of rat calvarial osteoblasts (RCO) *in vitro*. Their results showed that more cells attached and spread on the peptide-modified hydrogels than on the untreated ones, indicating that the FHRRIKA peptide addition improved the cell viability of the [P(NIPAAm-co-AAc)] hydrogels. Moreover, Cavalcanti-Adam *et al.* [122] investigated into the effects of the RGD peptide-modified silicone membranes on the osteogenic-related functions of osteoblast-like MC3T3-E1 cells *in vitro*. Their results showed that cells exhibited higher level of ALP activity after 8 days cultured on the RGD peptide-modified silicone membrane surface, indicating that the cells differentiated further into osteogenic ones better. Meanwhile, based on alizarin red staining and FTIR analysis, the cells cultured on the RGD-modified silicone membrane have better ability to generate biological apatite mineral deposition. Therefore, their results indicated that the RGD peptide addition significantly enhanced the osteogenic functions of the cells. In addition to the above-mentioned studies, researchers have investigated into metal biomaterials modified by ECM-derived peptides by cell culture *in vitro*. For example, Liu *et al.* [123] investigated into the osteogenic functions of preosteocyte MLO-A5 cells and mesenchymal cell (MSC) C3H10T1/2 on titanium surface modified by the P15 peptide with microscopies, real-time reverse transcription- polymerase chain reaction (qRT-PCR) analysis, western blotting and immunohistochemical analysis, etc., the results of which showed that the modification of titanium with P15 significantly increased not only the adhesion, spreading, and proliferation but also the maturation and osteogenic differentiation of the cells. Similarly, Reyes *et al.* [29] investigated into the osteogenic functions of bone marrow stromal cells on the titanium surfaces modified by GFOGER peptide *in vitro*. Their results showed that the cells cultured on titanium surfaces coated with the peptide achieved significantly higher expression of multiple osteoblast-specific genes (Fig. 3a), greater ALP activity (Fig. 3b), and biomineralized better (Fig. 3c) than those on untreated titanium surfaces.

**Figure 4.**
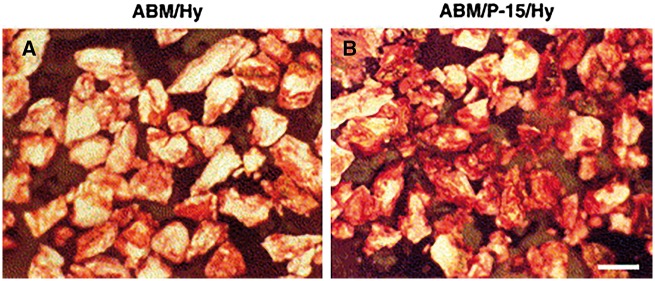
Alizarin red staining images of ABM/hy(a) and ABM/P-15/hy (B) cultured with HOS cells for 2 weeks. Bar =500 μm (adapted with permission from ref. [20]. Copyright 2003 Elsevier Ltd)

Besides the pure peptide-modified materials as described above, composites of them and other materials have been also widely researched by cell culture *in vitro* to evaluate the possibility of their application as bone repair materials. For instance, Nguyen *et al.* [20] modified anorganic bovine-derived mineral (ABM) with a typical ECM-derived peptide, P15, and then suspended them into hyaluronate (Hy) hydrogels, thereby preparing ABM/P15/Hy composites. Subsequently, they studied the effects of the P15 addition on the behaviours of osteoblast-like HOS cells *in vitro*. Their results showed that more cells adhered to ABM/P-15/Hy composites compared to ABM/Hy ones, and that the cells on ABM/P-15/Hy formed better surface coverage and had more stress fibers, suggesting that the P-15 addition promoted and strengthened cell adhesion. Most importantly, the cells cultured on ABM/P-15/Hy achieved significantly higher osteogenic gene expression of alkaline phosphatase and bone morphogenetic proteins, and biomineralized better (Fig. 4), indicating that the P-15 addition successfully enhanced the osteogenic differentiation and biomineralization of the cells.

#### BMPs-derived peptides modified materials

**Figure 5.**
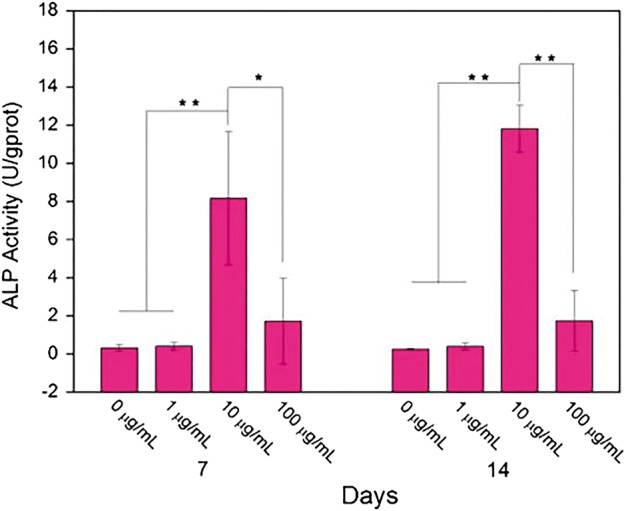
ALP Activity of MG-63 cells cultured on pure PAS and peptide-modified PAS with different incorporated concentrations for 7 and 14 days (adapted with permission from ref. [124]. Copyright 2016 Elsevier Ltd)

Up to now, many scholars have confirmed that BMPs-derived peptides modified materials could promote desired osteogenic functions of cultured cells *in vitro*. In their studies, single polymer modified by BMP-derived peptides is one kind of main material. For example, Luo *et al.* [124] investigated into behaviours of osteoblast-like MG-63 cells cultured on porous alginate scaffolds (PAS) modified by BMP-7-derived peptide, BFP-1, with scanning electron microscope (SEM), confocal laser scanning microscopy (CLSM), and ALP activity assay, etc., the results of which showed that the peptide introduction significantly increased not only the adhesion, spreading, proliferation, and aggregation but also osteogenic differentiation (Fig. 5) of the cells.

Besides single polymers, copolymers have also been used as substrates for the preparation of BMP-derived peptide-modified bone repair materials. For example, using PEG, amino acid units (ASP) and PLGA, Lin *et al.* [63] synthesized the PLGA-(PEG-ASP)_n_ copolymer, which was then modified by P24. Their results showed that P24/PLGA-(PEG-ASP)_n_ could improve better attachment of the bone MSCs and increase more significantly the expression of their osteogenic genes than PLGA-(PEG-ASP)_n_ and PLGA groups.

Moreover, composites modified by BMP-derived peptides have recently attracted more and more attention. For instance, Zhang *et al.*[60] investigated the effects of nHAC scaffolds modified by BMP-2-derived peptides, P17-BMP-2, on rabbit marrow stromal cells *in vitro*, the results of which showed that P17-BMP-2 compounded into the nHAC not only retained its activity, but also significantly upregulated the expression level ofosteogenic-related genes of the cells, such as OPN and OCN. Meanwhile, the cells could attach better on P17-BMP-2/nHAC than on nHAC. Moreover, Li *et al.* [125] *in vitro* studied true bone ceramics (TBC)/collagen I composites modified by another BMP-2-derived peptides, P24, by culturing bone marrow stromal cells. Based on SEM, energy dispersive X-ray spectroscopy (EDX), and X-ray diffraction (XRD) analysis, etc., they found that the P24 addition significantly enhanced the level of hydroxylapatite crystal mineralization with a Ca/P molar ratio of 1.63.

In addition to the above-mentioned studies, researchers have investigated into particles modified by BMP-derived peptides as a delivery system to find out their potentials as bone repair materials. For example, Bergeron *et al.* [126] compounded pBMP-9 peptide into collagen/45S5 Bioglass® microspheres for their controlled release, the results of which showed that the collagen/45S5 Bioglass^®^ group could release proteins more slowly than the pure collagen group (control). Moreover, the collagen/45S5 Bioglass^®^ microspheres containing the pBMP-9 peptide could induce the osteogenic differentiation of MC3T3-E1 preosteoblasts better than those compounded with rhBMP-2. Similarly, Zhou *et al.* [61] synthesized the residues 73-92 of BMP-2 covalently functionalized mesoporous silica (MSNs-pep) via an aminosilane linker. The cell viability of MSNs-pep was tested by bone MSCs exposure to different particle concentrations *in vitro*. The results revealed that the modified MSNs had better cytocompatibility, and that the cellular uptake efficiency of MSNs-pep was significantly larger than that of bare MSNs. Moreover, it was shown that the peptide addition significantly enhanced the osteogenic differentiation of the bone MSCs, based on the data of ALP activity, calcium deposition, and expression of bone-related proteins, etc.

#### Materials modified by other peptides

Compared to the materials modified by ECM or BMP-derived peptides, those modified by other active peptides were researched less *in vitro*. Recently, calcium phosphate minerals modified by other peptides have been used as coatings of bone repair materials. For example, Chen *et al.* [127] prepared OGP modified mixture of CaO and HA coatings on titanium substrates, and then investigated into the effects of the coatings on the behaviours of MSCs with XPS, SEM, and CLSM, etc., the results of which showed that the OGP addition significantly increased not only the adhesion and proliferation but also the maturation and osteogenic differentiation of the cells. Moreover, collagen matrix composites modified by self-assembly peptides have recently attracted more and more attention. For instance, Li *et al.* [108] developed RADA16-I peptide-modified demineralized bone matrix (DBM) material for bone repair, and studied them *in vitro* by culturing MSCs, which showed that the levels of expression of ALP, OCN, and Runx2 gene in DBM modified by RADA16-I were significantly higher than those in unmodified DBM at 14 days. Besides the above studies, biomacromoleculars modified by other active peptides have also researched *in vitro* to evaluate the potential of their application as bone repair materials. For instance, Suh *et al.* [128] prepared CPP peptide-modified a typical protein, PDZ-binding motif (TAZ), and then studied the effects of the CPP addition on the osteogenic differentiation of hMSCs. Their results showed that the cells cultured on the TAZ protein modified by CPP peptide achieved significantly higher expression of multiple osteoblast-specific genes (ALP, OCN, and Runx2), and biomineralized better than those on the untreated TAZ.

### *In vivo* evaluations

Currently, besides the *in vitro* studies, many researchers have investigated into peptide-modified materials by animal experiments *in vivo* to find out their potentials as bone repair materials, providing more direct data for their possible further clinical applications.

#### ECM-derived peptides modified materials

In recent years, many researchers have tried their best to heal bone defects of animals using calcium phosphate modified by ECM-derived peptides. For instance, Lindley *et al.* [129] created 4 mm diameter defects by drill bits in the tibiae of rabbits. Then, the animals were divided into four groups, which were respectively implanted with ABM/P-15/hyaluronate hydrogel, ABM/hyaluronate hydrogel, hyaluronate hydrogel alone, and nothing. Histomorphometric analyses showed that defects treated with ABM/P-15 had significantly larger areas of new bone formation than the other three groups at 2, 6, and 8 weeks after surgery. However, some other researchers got opposite outcomes of repairing bone defects with ABM/P-15. For example, Sarahrudi *et al.* [130] created 5 mm defects by high-speed oscillating saw in the femur of rabbits. Then, the animals were divided into two groups, which were respectively implanted with ABM/P-15 and nothing (control). Histomorphometry analyses showed that the ABM/P-15 groups have a smaller amount of new bone formation (1.56 ± 0.27 mm^2^) than the control group (2.5 ± 0.2 mm^2^) at 12 weeks after operation.

Beside the above studies, researchers have also investigated the osteogenic activity *in vivo* of collagen modified by ECM-derived peptides. For example, Egusa *et al.* [27] implanted atelocollagen sponge containing either 10 mg SVVYGLR peptide or PBS (as control) into the calvaria defects (5 mm in diameter and 0.5 mm in depth) of rats created by dental round burr. Their results showed that the number of osteoblasts in the SVVYGLR modified implants at 3 weeks after surgery was significantly higher than that in the control group. Meanwhile, newly formed blood vessels in the peptide-modified graft groups were more evident than those in the control group. Moreover, at five weeks after surgery, although both groups showed new bone formation in the cavity surrounding the sponge graft, more compact woven-like bone formed in animals treated with the atelocollagen sponge modified by SVVYGLR peptide.

In addition to pure peptide-modified materials as described above, composites containing them have been also researched *in vivo*. For example, Bitschnau *et al.* [131] prepared RGD-modified HA coatings on stainless steel K-wires, and then investigated into the effects of their implantation into the intramedullary canal of the rabbit tibia on the new bone formation and implant bone contact with quantitative and qualitative histology analysis, the results of which showed that RGD-HA and pure HA coated K-wires displayed higher new bone formation and implant bone contact than the uncoated ones after 12 weeks. There were no significant differences between the RGD-HA and the pure HA coated K-wires in new bone formation and implant bone contact after 4 and 12 weeks, the reason of which might be that the strong osteoconductive effect of the HA coating ‘‘over-whelms’’ the potential RGD effect.

#### BMPs-derived peptides modified materials

To date, many scholars have also studied the potential of *in vivo* repairing bone defects in animals of BMP-derived peptide-modified materials. For instance, Bergeron *et al.* [132] evaluated the bone repair ability of the pBMP-9 peptide bonded to type I collagen and chitosan by injecting them into mouse quadriceps. Histological analyses clearly demonstrated that more lamellar bone formed in animals treated with the chitosan modified by pBMP-9.

**Figure 6.**
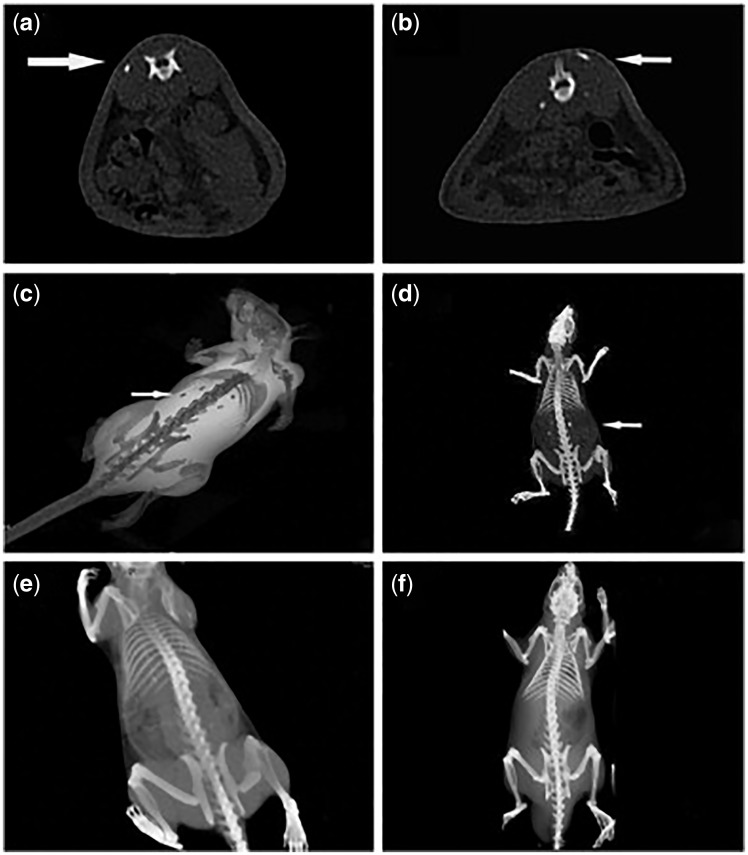
CT Images of P24/PLGA-(PEG-ASP)_n_ (a-d), PLGA-(PEG-ASP)n (e), and gelatin sponge (f) after implanted into the dorsal muscle of rats for 12 weeks after operation. Arrows indicated the new bone formation (adapted with permission from ref. [63]. Copyright 2010 Elsevier Ltd)

Besides single polymers, copolymers modified by BMPs-derived peptides have also been researched *in vivo*. For example, Lin *et al.* [63] respectively implanted P24/PLGA-(PEG-ASP)_n_, PLGA-(PEG-ASP)_n_ and gelatin sponge into the dorsal muscle of rats. Radiographic examination showed that the P24/PLGA-(PEG-ASP)_n_ group had block-like shadows on the CT image at 12 weeks after the surgery (Fig. 6a–d) while no high-density shadows were observed in groups of PLGA-(PEG-ASP)_n_ (Fig. 6e) and gelatin sponge (Fig. 6f). Subsequently, histological examinations confirmed that new bone formed at the subcutaneous layers, where high-density shadows were shown by the CT scans.

**Figure 7.**
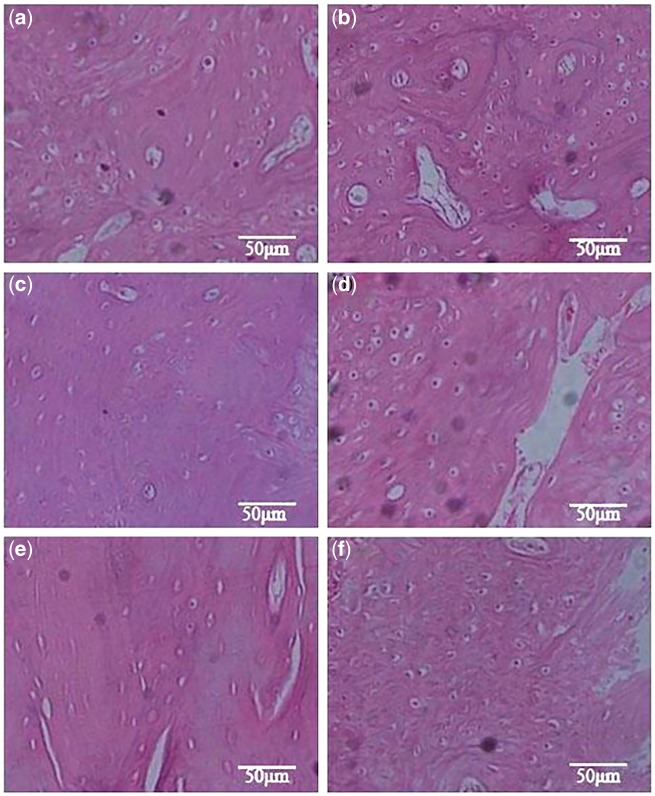
The histological images of the implanted materials at two time points: (a) P24/TBC/collagen I, (c) TBC/collagen I, (e) TBC at 8 weeks; (b) P24/TBC/collagen I, (d) TBC/collagen I, and (f) TBC at 12 weeks (magnification: 200×) (adapted with permission from ref. [125]. Copyright 2010 Elsevier Ltd)

Moreover, BMP-derived peptide-modified composites have also been tried to repair bone defects *in vivo*. For example, Li *et al.* [125] created 10 mm unilateral segmental bone defect with burr drill in rabbit radius. Then, the animals were divided into three groups, which were respectively implanted with P24/(true bone ceramics) TBC/collagen I composite (Group A), TBC/collagen I composite (Group B), and TBC (Group C). Based on histological examination at 8 and 12 weeks after surgery, more newly formed bone was observed in Group A (Fig. 7a and b) than those in Group B (Fig. 7c and d) and Group C (Fig. 7e and f). In addition, Li *et al.* [133] implanted nHAC/PLLA containing either P24, rhBMP-2, or nothing into the cranial bone defects (5 mm diameter) of rats created by trephine drill. The results of the radiographic and three-dimensional CT evaluations and the histological examinations showed that the P24 addition much more significantly enhanced the bone defect repair effectiveness.

#### Materials modified by other peptides

At present, *in vivo* bone repair ability of materials modified by other active peptides, besides ECM or BMPs-derived ones, have been also studied. For example, Sheller *et al.* [134] prepared PLGA microspheres modified by TP508 peptide, and implanted them into the defects of rabbit forelimbs (0.5 cm in length) to evaluate their bone repair ability. The radiographs showed a significantly higher degree of bone repair in the animals treated with PLGA microspheres modified by the peptide. Three-dimensional synchrotron tomography showed that the new bone in animal treated with the peptide-modified PLGA microspheres had a less porous surface appearance and more open marrow spaces than that in the animal treated with unmodified PLGA microspheres, indicating that the implantation of the peptide-modified material could get a better result of bone remodeling. In addition, Ma *et al.* [135] created 1.5 cm defects by saw in the rabbit radius. Then, the animals were divided into two groups, which were respectively implanted with OGP/PLGA scaffold and bare PLGA scaffold. Radiographic images and histomorphological analysis showed that more new bone formed in animals treated with the PLGA scaffolds modified by the OGP peptide.

Moreover, hydrogels modified by other peptides have been researched *in vivo*. For instance, Jung *et al.* [136] synthesized the polyethylene glycol (PEG) hydrogel, which was then modified by PTH_1-34_ and RGD peptides. In their study, circumferential bone defects were created in foxhound mandibular premolar, and then the animals were randomly divided into four groups, which were respectively implanted with PEG containing PTH_1-34_ and RGD peptides, PEG, autogenous bone, and nothing (control). Histomorphometric analysis showed that newly formed bone in the peptide-modified graft groups were much more evident than that in the groups of PEG or control at 4 and 12 weeks after surgery. Furthermore, it was indicated that the peptide-modified materials could g*et al*most the same bone repair effectiveness compared with autogenous bone.

## Conclusion and perspective

In this article, recent studies on bioactive peptide-modified materials for bone tissue repair have been generally reviewed. Currently, many kinds of peptides, including ECM-derived ones, BMPs-derived ones, etc., have been developed and investigated as valid candidates for bone healing. These peptides can activate some specific signalling pathways that control osteogenic-related cellular functions. Meanwhile, a lot of studies have been launched to modify bone repair materials with these peptides with many different methods, such as electrodeposition, covalent immobilization, physical adsorption, etc. In combination with the peptides, the materials have been generally shown to possess enhanced osteogenic ability, presenting to induce osteogenic-related cellular responses and further promote new bone formation and osseointegration. Generally speaking, the recent related studies have fully suggested that the modification of bone repair materials with osteogenic-related peptides provide promising strategies for the development of bioactive materials and substrates for enhanced bone regeneration and the therapy of bone tissue diseases.

Although great achievements have been got, there is still a lot of work to do. Firstly, more detailed systematic studies to figure out more specific characteristics and potential functions of each related peptide are necessary. Secondly, more satisfactory techniques need to be developed to prepare the bioactive peptide-modified materials for different applications, in which the amount of loaded peptides can be flexibly controlled. Furthermore, the loaded peptides should be able to release at a controllable rate. Thirdly, since it has been shown that the preparation method of the peptide-modified materials has significant effects on the bioactivity of the peptides, the systematic investigations into the possible influential mechanisms should be launched. Especially, although there have been already many methods to covalently bond peptides to materials, hardly any publication on how the different covalent bonding methods affect the bioactivity of the same peptide can be found. Fifthly, there are many factors that influence the function realization of the peptide-modified materials, among which degradation of the substrate materials is a crucial one. Future studies on this aspect are very necessary. Sixthly, for the bone repair materials, it is well recognized that their positive effects on the biomineralization is one of most important evaluation standards of their quality. However, the current investigations into the effects of peptide-modified materials for bone repair on the biomineralization *in vitro* and *in vivo* are very inadequate. Finally, the efforts to ensure the loaded peptides on the bone repair materials to entirely reach the targeted sites to utmostly promote new bone formation still need to be made in future studies.

## Funding 

National Natural Science Foundation of China (Nos. 31370959, 11421202 and 61227902), Fok Ying Tung Education Foundation (No. 141039), Beijing Nova Programme Interdisciplinary Cooperation Project (No. xxjc201616), Key Laboratory of Advanced Materials of Ministry of Education of China (Tsinghua University), International Joint Research Center of Aerospace Biotechnology and Medical Engineering, Ministry of Science and Technology of China, and the 111 Project (No. B13003).

*Conflict of interest statement*. None declared.
